# Improving Screening for Latent Tuberculosis Infection in a Student-run Free Clinic

**DOI:** 10.7759/cureus.2488

**Published:** 2018-04-16

**Authors:** Ambika Anand, Courtney Wagner, Steve S Kong, Elliot Griffith, Punnavit Harimtepathip, Kathryn K Baker, Stephen Rineer, Judith Simms-Cendan, Magdalena Pasarica

**Affiliations:** 1 University of Central Florida College of Medicine, Orlando, USA; 2 Medical Education, University of Central Florida College of Medicine, Orlando, USA

**Keywords:** tuberculosis, ltbi, screening, preventative medicine, student-run free clinics

## Abstract

Introduction

Latent tuberculosis infection (LTBI) screening with targeted treatment has been successful in eradicating tuberculosis (TB) as an endemic infection in the United States. The Centers for Disease Control and Prevention (CDC) recommends screening for high-risk patients. The aim of this study was to increase LTBI screening, detection, and treatment in our student-run free clinic while providing an innovative platform for education in primary care topics.

Methods

A questionnaire for screening for LTBI was adapted from CDC guidelines. Medical students and providers received education on the screening process and administered questionnaires to patients. We analyzed the rate of performed LTBI screening, the rate of diagnostic testing for patients with positive screening, and the feasibility of implementing a preventive screening initiative.

Results

Fifty-two patients completed primary care visits. Forty patients were screened for LTBI. Of those screened, 42.5% were positive for the screening. Of those with positive screening, 70.6% were followed up via diagnostic testing, with the rest of them being lost for follow-up due to not attending the clinic for care.

Conclusions

This educational intervention combined with a screening tool was effective in increasing LTBI screening rates amongst patients in a student-run free clinic.

## Introduction

In the United States (US), latent tuberculosis infection (LTBI) screening with targeted treatment has been successful in eradicating tuberculosis (TB) as an endemic infection. The Centers for Disease Control and Prevention (CDC) recommends that patients should be asked about their risk factors for TB and have tuberculin skin testing (TST) or interferon-gamma release assay (IGRA) tests if risk factors are present [1]. Early intervention prevents the patient from developing active TB or spreading infection to the community. Identifying subpopulations that would benefit from screening would help not only that specific population but also the broader population.

The Keeping Neighbors In Good Health Through Service (KNIGHTS) Clinic is run by medical students from the University of Central Florida College of Medicine and pharmacy students from the University of Florida College of Pharmacy. It is a student-run free clinic that provides primary care and specialty care services to underserved patients located in Orange County, Florida. Orange County was ranked third in the state of Florida for cases of TB and ninth for incidence rate [2]. Orange County had higher incidence rates of preventable hospitalizations from non-pulmonary TB than those for the state of Florida as a whole [3].

Outside of the US, the literature suggests educational programs and screening protocols help increase identification of active TB disease [4]. Student-run free clinics may play a role in implementing such programs for improved patient outcome and establishing innovative learning of primary care topics.

We aimed to improve the screening and detection of LTBI in our patient population by initiating a quality improvement project at KNIGHTS Clinic to include education of clinic volunteer students and providers and a screening questionnaire. Our aim was to increase LTBI screening, detection, and treatment in our clinic while providing an innovative platform for education in primary care topics.

## Materials and methods

A screening questionnaire for LTBI was adapted from the CDC (Table 1) [1]. Medical providers and volunteer medical students received education on screening during a board meeting and subsequent briefings before each clinic. Initially, screening was tasked to a team of students called “patient educators” who typically provide counselling on lifestyle modification. This was cumbersome for the patient educators and more feasible for the primary health-care provider team (medical student volunteers and their attending physician) to administer the questionnaire.

**Table 1:** Sample of LTBI screening questions adapted from CDC guidelines. CDC: Centers for Disease Control and Prevention; LTBI: Latent tuberculosis infection; TB: Tuberculosis; HIV: Human immunodeficiency virus.

Questions in the electronic medical record system	Answer options
The patient spent time with someone who has TB disease	Yes, No, Unknown
Has HIV infection or another medical problem that weakens the immune system	Yes, No, Unknown
Has symptoms of TB disease	No, fever, night sweats, cough, weight loss, other
Is from a country where TB disease is common	No, Latin America, Caribbean, Africa, Asia, Eastern Europe, Russia, other
Lives or works somewhere in the US where TB disease is more common	No, homeless shelter, prison or jail, nursing home, other
Uses illegal drugs	Yes, No, Unknown

The completed questionnaires were originally scanned into the patient record, but eventually were incorporated directly into the electronic medical record (EMR) as its own assessment (Table 1), warranting further instruction for the primary health-care provider team on proper documentation of screenings within the EMR. Patients with any risk factors for LTBI were counselled for further evaluation via TST or IGRA.

Data was collected via chart review and included patient age, gender, ethnicity, clinic date, screening completion, positive risk factors, diagnostic testing (TST, IGRA, or chest radiograph) with results, and treatment. For patients lost to follow-up, chart review was conducted to determine reasons behind lack of follow-up. We analyzed the rate of LTBI screening, type of diagnostic TB testing in patients with risk factors, and patients’ presenting risk factors. Data was analyzed after 14 months of project implementation.

## Results

Despite the known TB statistics of Orange County and the knowledge that many patients have contact with regions endemic for TB, patients were rarely assessed for LTBI during standard visits. As such, during the three months preceding the intervention, 20 patients received medical care in the clinic without any documented screening for TB risk factors.

**Figure 1:**
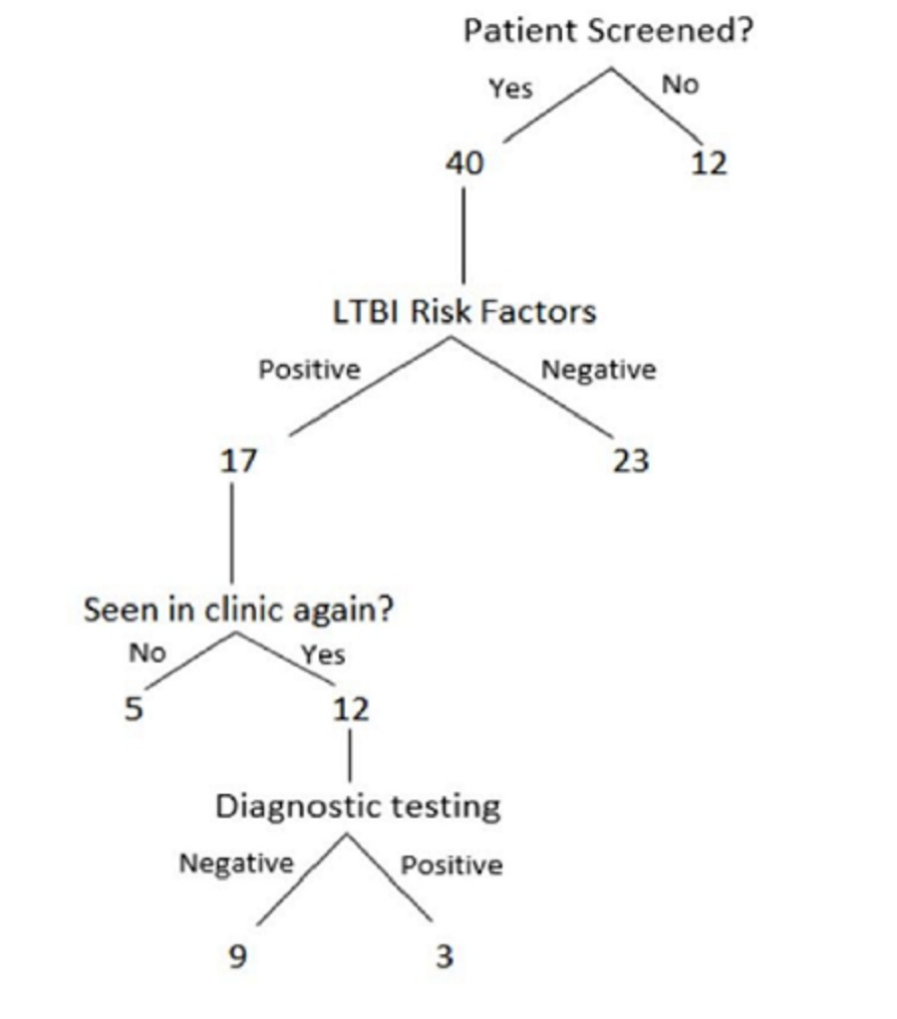
Diagram of screening events. LTBI: Latent tuberculosis infection.

After we implemented the intervention (from December 2015 to February 2017), 52 patients completed primary care visits, and 76.9% of patients were screened for LTBI. Of those who received screening for LTBI, 42.5% were positive. Most positive screening patients (30%) were at risk because they emigrated from countries located in TB endemic regions. Other identified risks are described in detail in Table 2. Out of those with positive risk factors, 70.6% patients received diagnostic TB testing. The remaining patients did not receive diagnostic TB testing due to no longer being patients of the clinic, most likely due to obtaining medical insurance (note that our clinic can only see patients without any kind of medical insurance). Of the 12 patients followed up, three had a positive diagnosis and were treated according to recommended CDC guidelines. The sequence of screening results is presented in Figure 1.

**Table 2:** Risk factors present in our patient population. The table shows the breakdown of risk factors found among the patients who were screened from the Latent Tuberculosis Infection (LTBI) questionnaire implemented by our student-run free clinic. TB: Tuberculosis; PPD: Purified protein derivative; BCG: Bacillus Calmette–Guérin.

Percentage of screened patients with associated risk factors	
Emigrated from endemic region	30.0%
Symptoms of active TB	10.0%
Recent exposure to TB	2.5%
History of positive PPD or Quantiferon Gold	7.5%
Diabetes mellitus	7.5%
Recent travel to the endemic region	5.0%
Born in foreign country	5.0%
Illegal drug use	2.5%
History of BCG vaccine	5.0%
Immunosuppressive therapy	2.5%
History of latent TB	0.0%
Immunodeficiency	0.0%

## Discussion

During the study period, the majority of our patients receiving care from our free care clinic were screened for LTBI. 5.4% of those who were screened tested positive for LTBI. This is comparable to the national estimate of LTBI in the US, which the United States Preventive Services Task Force recommendation statement on LTBI screening in adults places at 4.7% to 5.0% [5]. The increased rate of screening and ease of implementation demonstrate high feasibility and usefulness of a questionnaire for this public health intervention. Using the screening questionnaire, we were able to separate the high-risk population from the low-risk population to prevent unnecessary diagnostic testing in the low-risk population [5]. Our targeted testing resulted in reduced testing cost as well as reduced unnecessary healthcare burden to patients.

Limited literature exists focusing on US-based primary care and screening for LTBI. A recent analysis by the CDC showed that TB rates in the US had remained the same from 2013 to 2015, indicating that TB rates had become stagnant, rather than declining, in the US for the first time in 20 years [5]. This report suggests there is a continued need for LTBI screening and testing. In a 2007 randomized controlled trial of UK-based general practices, implementation of an educational program greatly increased TB screening rates [4].

Screening for individuals with high risk of LTBI reactivation and early treatment is the first main pillar highlight by the World Health Organization End-TB strategy aimed at the elimination of TB [6]. An increase in the integration of routine LTBI screening surveys in clinics across the country will identify more individuals who are at risk of having LTBI. The subsequent early treatment of these individuals may perhaps provide a solution to the problem of stagnant TB rates in recent years. A future direction of this study would be to track the initiation and completion of treatment in the population that tested positive in the initial LTBI screening.

The high rate of screening and ease of implementation demonstrated by our study shows the utility of a questionnaire for public health intervention. LTBI questionnaire screening is easily reproducible due to the small amount of required resources. EMR integration allowed for better tracking. A limitation of the study is the small sample size due to the smaller clinic capacity. Future studies could include the entire clinic population for a more quantitative analysis. The study is based on a low-income population, which limits the generalizability. Increasing integration of routine LTBI screening surveys in clinics across the country would ideally start with other student-run clinics and expand to help identify more individuals at risk of having LTBI, prompt early treatment, and help solve the problem of stagnant TB rates in recent years.

## Conclusions

In conclusion, we found that combining educational intervention with an effective screening tool increased LTBI screening rates amongst our patients. As medical students, we were able to implement changes at our student-run free clinic to make an impact on public health and safety, while improving our education in a critical issue for primary care. We will continue to screen for LTBI, optimize these interventions, and analyze TB screening rates.
